# Role of LRRK2 in the regulation of dopamine receptor trafficking

**DOI:** 10.1371/journal.pone.0179082

**Published:** 2017-06-05

**Authors:** Mauro Rassu, Maria Grazia Del Giudice, Simona Sanna, Jean Marc Taymans, Michele Morari, Alberto Brugnoli, Martina Frassineti, Alessandra Masala, Sonia Esposito, Manuela Galioto, Cristiana Valle, Maria Teresa Carri, Alice Biosa, Elisa Greggio, Claudia Crosio, Ciro Iaccarino

**Affiliations:** 1 Department of Biomedical Sciences, University of Sassari, Sassari, Italy; 2 UMR-S1172, Jean-Pierre Aubert Research Center (Inserm – Université de Lille – CHRU de Lille), Lille, France; 3 Department of Medical Sciences, University of Ferrara, Ferrara, Italy and National Institute for Neuroscience, Ferrara, Italy; 4 Fondazione Santa Lucia, IRCCS, Rome, Italy; 5 Institute of Cell Biology and Neurobiology, IBCN, CNR, Rome, Italy; 6 Department of Biology, University of Rome Tor Vergata, Rome, Italy; 7 Department of Biology, University of Padova, Padova, Italy; Van Andel Institute, UNITED STATES

## Abstract

Mutations in LRRK2 play a critical role in both familial and sporadic Parkinson’s disease (PD). Up to date, the role of LRRK2 in PD onset and progression remains largely unknown. However, experimental evidence highlights a critical role of LRRK2 in the control of vesicle trafficking that in turn may regulate different aspects of neuronal physiology. We have analyzed the role of LRRK2 in regulating dopamine receptor D1 (DRD1) and D2 (DRD2) trafficking. DRD1 and DRD2 are the most abundant dopamine receptors in the brain. They differ in structural, pharmacological and biochemical properties, as well as in localization and internalization mechanisms. Our results indicate that disease-associated mutant G2019S LRRK2 impairs DRD1 internalization, leading to an alteration in signal transduction. Moreover, the mutant forms of LRRK2 affect receptor turnover by decreasing the rate of DRD2 trafficking from the Golgi complex to the cell membrane. Collectively, our findings are consistent with the conclusion that LRRK2 influences the motility of neuronal vesicles and the neuronal receptor trafficking. These findings have important implications for the complex role that LRRK2 plays in neuronal physiology and the possible pathological mechanisms that may lead to neuronal death in PD.

## Introduction

Mutations in the leucine-rich repeat kinase 2 gene (LRRK2, PARK8) are the most frequent genetic causes of Parkinson’s disease, reaching up to 40% in some ethnic groups, Ashkenazi Jewish and North African Arab Berbers [1]. These mutations cause late-onset, autosomal dominant PD that is clinically and neuropathologically indistinguishable from idiopathic forms [2, 3]. LRRK2 is a member of Roco superfamily proteins, a novel multi-domain family of Ras-like G-proteins. LRRK2 is composed of different functional and structural domains: armadillo repeats (ARM), ankyrin repeats (ANK), leucine-rich repeats (LRR), Ras of complex (Roc), C-terminal of Roc (COR), kinase and a WD40 domains [4]. Up to date, the PD pathological mutations have been identified around the central catalytic core of the protein: two mutations in the Roc domain (N1347H and R1441C/G/H/S), one in the COR domain (Y1699C) and two in the kinase domain (G2019S and I2020T). In addition, two risk factor mutations for sporadic PD were identified, respectively in the COR domain (R1628P) and in the WD40 repeats (G2385R) [4].

Despite the apparent clinical association between LRRK2 mutations and PD, it remains enigmatic how LRRK2 pathological mutations may contribute to disease onset and progression. Different experimental results suggest an important role of LRRK2 in the control of vesicle trafficking, and alteration in synaptic vesicle trafficking seems a common theme in PD pathogenesis [5, 6]. Moreover, many LRRK2 protein interactors belong to protein families involved in vesicle trafficking regulation inside the cells (among them Rab5 [7], Rab7 [8], Rab7L [9, 10], Sec16A [11], a subset of Rabs [12], endoA [13]) or in cytoskeleton dynamics that in turn may modulate vesicle trafficking [14–17].

In neurons, the vesicle trafficking controls fundamental physiological functions such as neurotransmitter or protein release and uptake, localization of membrane receptors, changes in plasma membrane composition and, not least, organelle biogenesis. LRRK2 has been implicated in the regulation of receptor trafficking: DRD2 protein level is elevated in LRRK2 over-expressing mice [18], loss of LRRK2 impairs the activity-dependent targeting of glutamate receptors into the cell/synapse surface [11], LRRK2 over-expression, mostly the pathological mutants, alters the level of epidermal growth factor receptor (EGFR) on cell membrane and its degradation pathway [19].

We have previously shown that the expression of disease-associated LRRK2 mutants lead to alteration of DRD1 trafficking both in animal and cellular models. In particular, expression of G2019S LRRK2 determines an increase in DRD1 on the membrane that parallels a decrease in the vesicle pool [20]. The neurotransmitter receptor level on plasma membrane is determined by the protein arriving on the cell surface from Golgi/exocytic pathways, the protein leaving the surface via the endocytic pathway, and eventually the receptor recycling to plasma membrane from the intracellular endosomal pools. Consequently, many different molecular pathways could be responsible for the DRD1 trafficking/localization alteration that we observe in transgenic mice. Based on these considerations, we investigated the molecular mechanism behind LRRK2 action on DRD1 and extended our analysis to other members of the dopamine receptor family. DRD1 and DRD2 are the most abundant dopamine receptors in the CNS and belong to two different receptor classes: D1-class dopamine receptors (D1 and D5) or D2-class dopamine receptors (D2, D3, and D4) [21, 22]. In addition, alternative splicing of Drd2 gene generates two major D2 dopamine receptor variants: DRD2S (D2-short) and DRD2L (D2-long) [23, 24]. Importantly D1- and D2-class dopamine receptors differ in structural, pharmacological, biochemical properties, as well as in localization and internalization mechanisms [21]. Here we provide evidence that LRRK2 plays an important role in the control of DRD1 and DRD2 trafficking both in cellular and animal models.

## Results

### LRRK2 G2019S alters the internalization of dopamine D1 receptor

**Fig 1:**
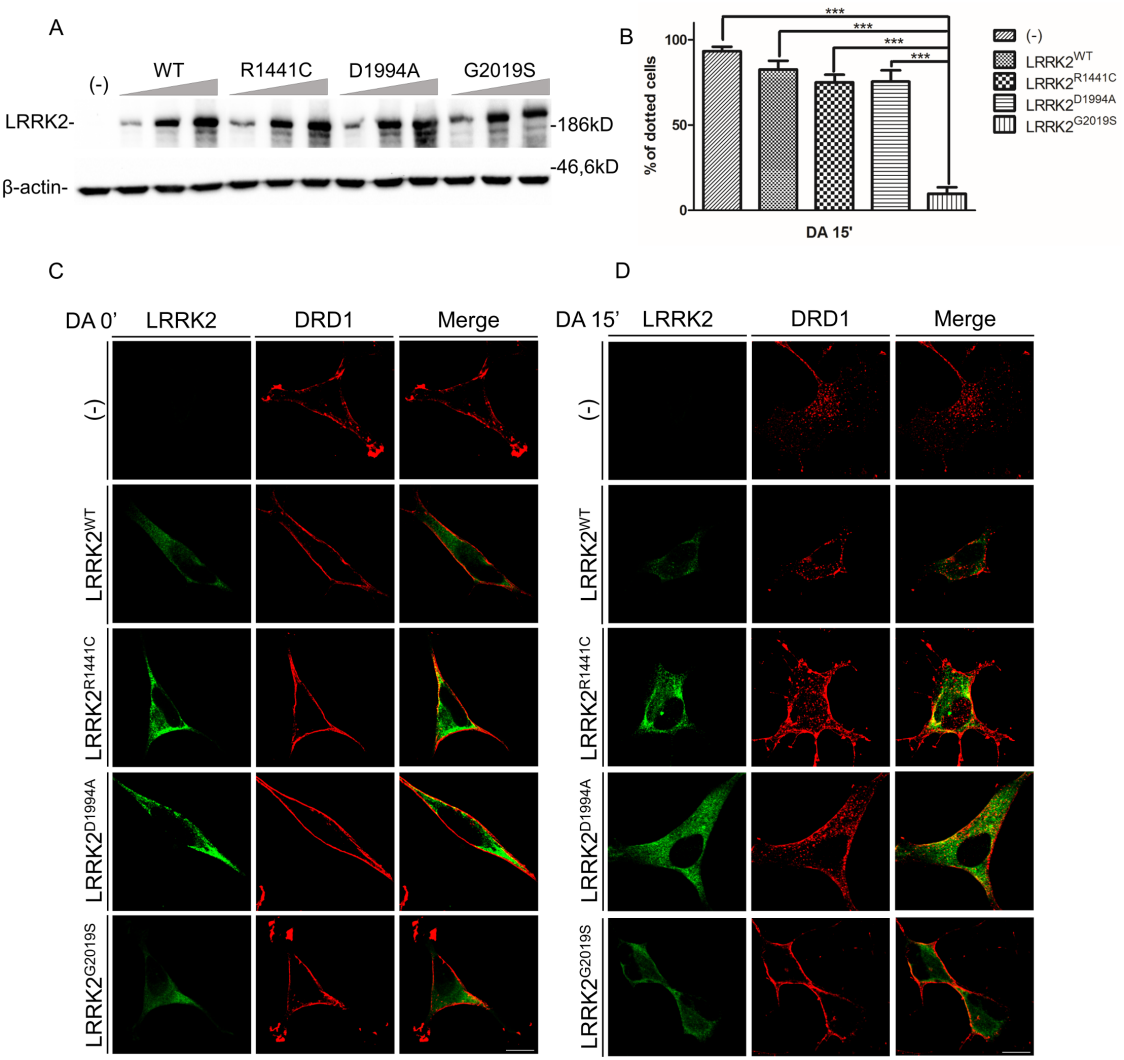
Analysis of DRD1 internalization upon dopamine treatment in SH-SY5Y-DRD1 cells untransduced or transduced by WT or different LRRK2 mutants. **(A)** Evaluation of LRRK2 expression level upon recombinant adenovirus transduction in SH-SY5Y cells. SH-SY5Y were transduced by increasing amount recombinant adenoviruses and analysed 48 hours after transduction. Cell lysates were subjected to reducing SDS-PAGE and western blot. The anti-LRRK2 antibody (MJFF2 c41-2) was used to visualize LRRK2 expression level and β-actin serves as controls for equal loading of samples. **(C and D)** DRD1 localization at basal conditions (C) and upon 15 minutes (D) of dopamine treatment of SH-SY5Y-DRD1 cells transduced by the different recombinant adenovirus for 48h. After agonist treatment the cells were fixed and incubated by the different primary antibodies (anti-FLAG for DRD1 and anti-LRRK2 (MJFF2) for LRRK2) and with Alexa647-conjugated secondary antibody (red) or Alexa488-conjugated secondary antibody (green) for DRD1 or LRRK2 respectively. Scale bars = 10μm. **(B)** Quantification of data obtained in (D) using different LRRK2 mutants upon 15' of dopamine treatment. The data represent the mean ± SD. ***p<0,001. One-way ANOVA and Bonferroni post test were used.

To investigate the role of LRRK2 in the regulation of dopamine receptor trafficking we generated and characterized (i) recombinant adenovirus for the expression of different LRRK2 isoforms (Fig 1A) and (ii) two different SH-SY5Y stable cell lines expressing either DRD1 or DRD2L (long isoform) both bearing a C-terminal 3X-FLAG tag (see below).

Using these molecular tools, we analysed the internalization of D1 receptor upon dopamine treatment in SH-SY5Y-DRD1 cells by confocal microscopy, in the presence or absence of the different LRRK2 isoforms. LRRK2 expression was obtained by transduction of recombinant adenovirus for WT or the pathological mutant G2019S LRRK2. As shown in Fig 1C (left panel), the receptor is mainly localized on the cell membrane in SH-SY5Y-DRD1 at basal conditions, both in the absence or presence of wild type and mutant LRRK2. However, DRD1 is rapidly internalized upon 5 minutes of dopamine treatment, as shown by the presence of red dots inside the cells (S1A Fig). On the contrary, the presence of mutant G2019S LRRK2 impairs the receptor internalization. This effect becomes even more evident after 15 minutes of dopamine treatment (Fig 1D). After 1 hour of dopamine treatment some red dots are still visible in cells transduced by G2019S LRRK2 (S1B Fig). No significant difference, compared to WT LRRK2, on D1 internalization was observed in the presence of mutant R1441C or the dead kinase D1994A LRRK2 (Fig 1B and 1D). Moreover, the DRD1 trafficking alteration due to G2019S expression seems to be independent of LRRK2 kinase activity since pre-treatment with a LRRK2 kinase inhibitor (CZC-25146) does not significantly mitigate the G2019S LRRK2 effect (S2A and S2B Fig)

**Fig 2:**
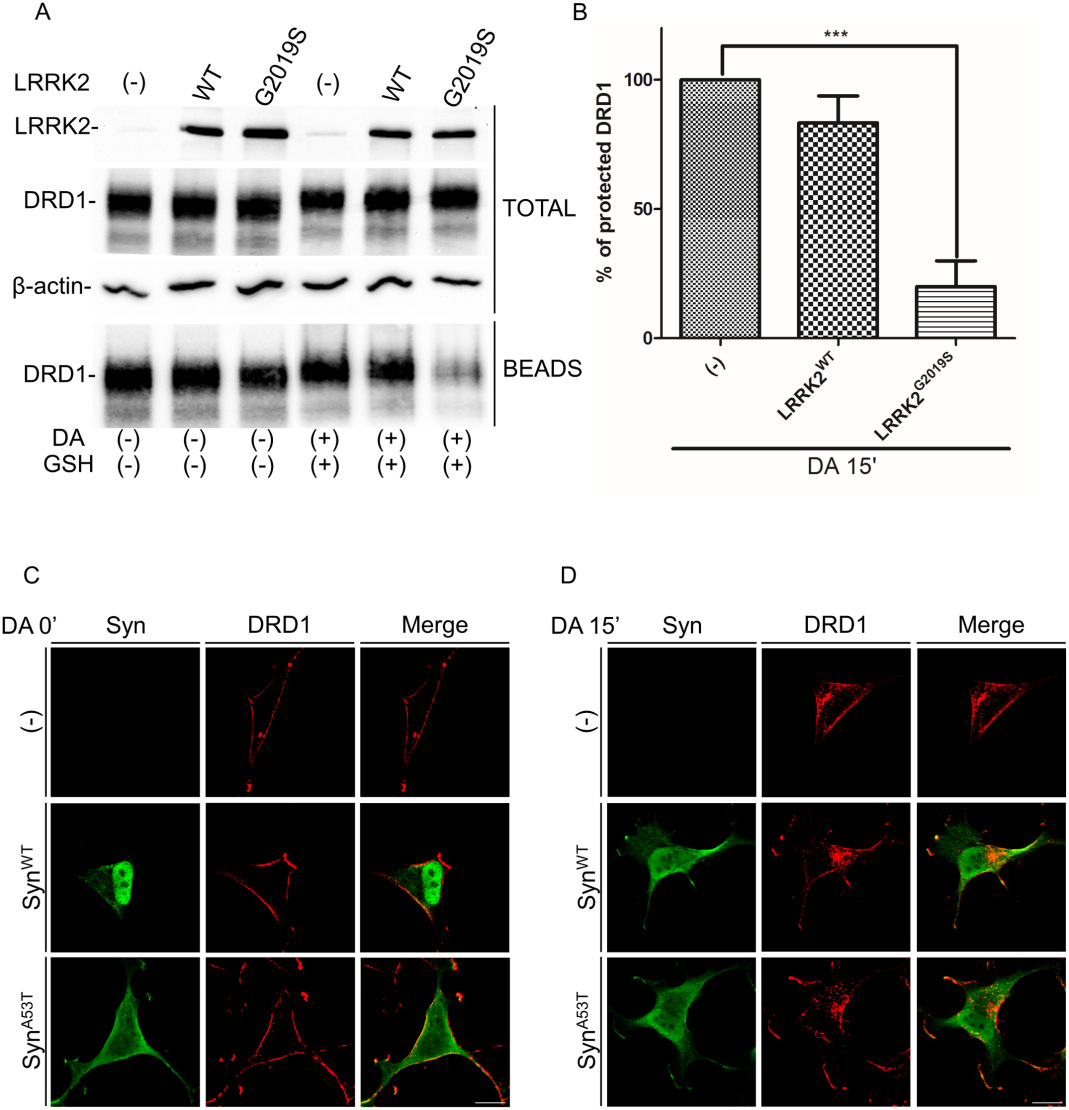
Evaluation of DRD1 intracellular and extracellular level by BPA upon dopamine treatment in SH-SY5Y-DRD1 cells untransduced or transduced by WT or G2019S LRRK2. **(A)** Cells stably expressing FLAG-tagged DRD1 were transduced by the different LRRK2 isoforms. 48h after transduction the cell membrane protein were labeled by biotin. After 15 minutes of dopamine treatment the cell surface-biotinylated receptors were stripped and the cell lysates subjected to purification by streptavidin-agarose. Total and immunoprecipitated (beads) proteins were visualized by western blot using specific antibody for the indicated proteins. β-actin serves as controls for equal loading of samples. **(B)** Densitometric analysis of data obtained in (A) nomalized by the untransduced cells. The data represent the mean ± SD. ***p<0,001. One-way ANOVA and Bonferroni post test were used. **(C and D)** DRD1 localization at basal conditions (C) and upon 15 minutes (D) of dopamine treatment of SH-SY5Y-DRD1 cells transduced or not by alpha-synuclein WT or A53T. After agonist treatment the cells were fixed and incubated with the different primary antibodies (anti-FLAG for DRD1 and anti-synuclein (Millipore) for alpha-synuclein) and with Alexa647-conjugated secondary antibody (red) or Alexa488-conjugated secondary antibody (green) for DRD1 or synuclein respectively. Scale bars = 10μm.

The reduced internalization of DRD1 observed in the presence of G2019S LRRK2 even at early time points of dopamine treatment indicates an alteration of endocytosis rather than an increase in recycling/exocytosis pathway. To get a quantitative evaluation on the alteration of DRD1 trafficking in the presence of G2019S LRRK2 we performed a biotin degradation protection assay (BPA) [25]. As illustrated in Fig 2A and 2B the level of DRD1 internalized upon 15 minutes of dopamine treatment is strongly reduced in G2019S LRRK2 transduced cells compared to untransduced cells. It is interesting to note the slight but significant shift in DRD1 molecular weight upon agonist treatment. In fact, in absence of any phospho-specific antibody for DRD1, the DRD1 shift has been largely used to measure the receptor phosphorylation/activation state [26].

Since different experimental evidence [27] suggests a potential role of synuclein in the control of vesicle trafficking, we have analyzed the possible effect of synuclein expression on DRD1 trafficking upon dopamine treatment in the same experimental conditions. As shown in Fig 2C and 2D no significant effect is detectable in the presence of either WT or pathological mutant A53T synuclein transduced by recombinant adenovirus.

### LRRK2 alters the dopamine D2 receptor trafficking

While, as expected, DRD1 is largely localized on the cell membrane, instead DRD2L is partially present inside SH-SY5Y cells, likely in vesicle structures and in the Golgi apparatus (see below). This is consistent with reports from other groups using similar DRD2L stable clones [28, 29]. Moreover, in SH-SY5Y cells, the dopamine receptor trafficking differs between the different subtypes. DRD1 is recycled back to the plasma membrane while DRD2 is mainly degraded after receptor internalization upon agonist treatment [25].

**Fig 3:**
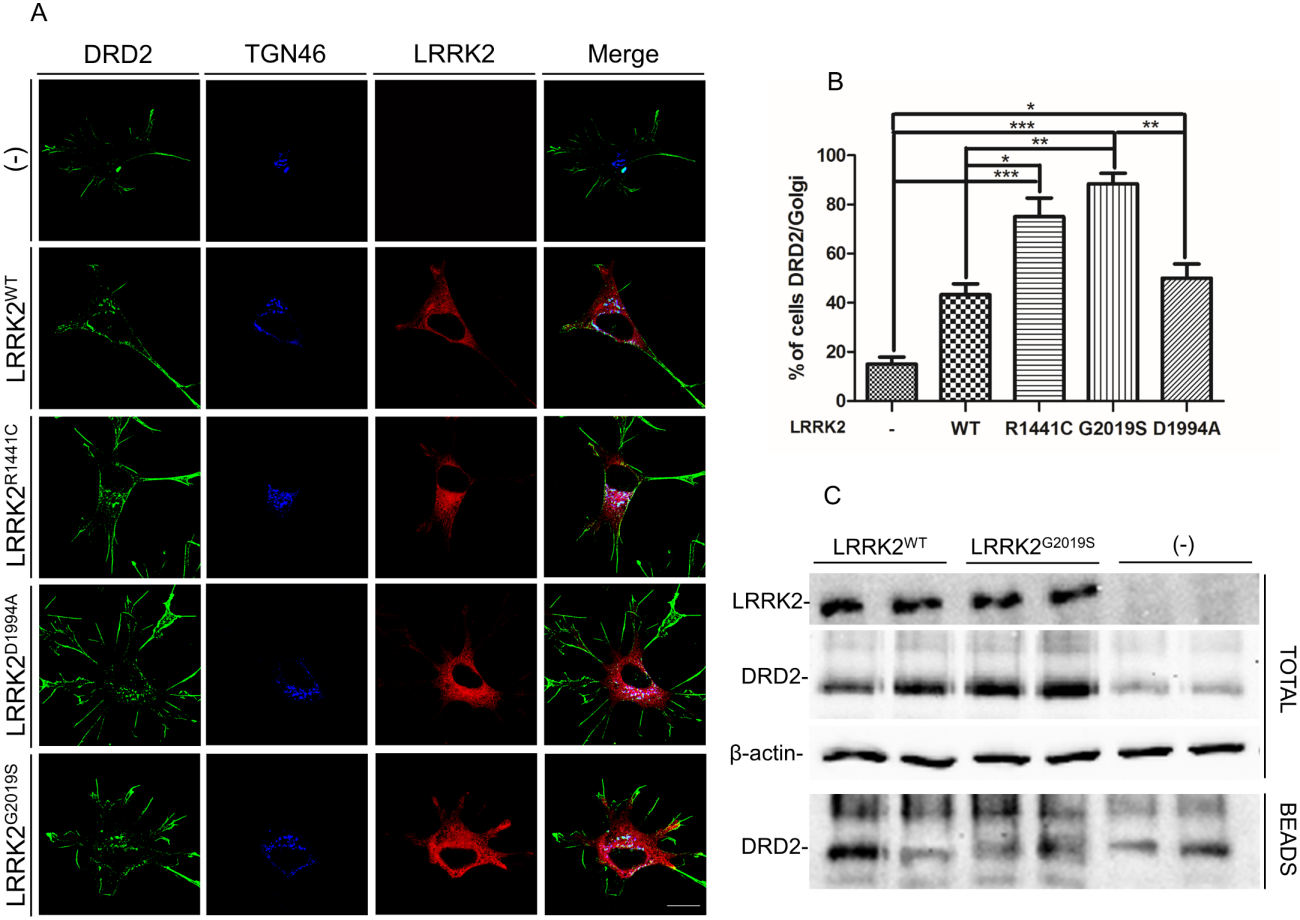
Analysis of LRRK2 effect on DRD2 cell localization. **(A)** SH-SY5Y cells stably expressing FLAG-tagged DRD2 were transduced by WT or different LRRK2 mutants. 48h after transduction, the cells were fixed and incubated with the different primary antibodies (anti-FLAG for DRD2 and anti-LRRK2 antibody (MJFF2 c41-2) for LRRK2 and anti-TGN46 for trans-Golgi staining). Scale bars = 10μm. **(B)** Quantification of data obtained in (A) and using different LRRK2 mutants. The data represent the mean ± SD. *p<0,05; **p<0,01; ***p<0,001. One-way ANOVA and Bonferroni post test were used. **(C)** Cells stably expressing FLAG-tagged DRD2 were transduced by the different LRRK2 isoforms. 48h after transduction the cell membrane protein were labeled by biotin. After 15 minutes of dopamine treatment the cell surface-biotinylated receptors were stripped and the cell lysates subjected to purification by streptavidin-agarose. Total and immunoprecipitated (beads) proteins were visualized by western blot using specific antibody for the indicated proteins. β-actin serves as controls for equal loading of samples.

First of all, we have evaluated possible alteration in DRD2 localization in the presence of LRRK2 over-expression. Stable SH-SY5Y-DRD2 cells were transduced by the different LRRK2 isoforms and DRD2 cellular distribution was analyzed 48 hours after transduction. As shown in Fig 3A and 3B, the over-expression of LRRK2 pathological mutants determines a significant accumulation of DRD2 into the cells compared to LRRK2 WT or dead kinase and even more compared to untransduced cells. Co-staining with a trans Golgi specific marker (TGN46) reveals that intracellular DRD2 is mainly localized into the Golgi apparatus (3A-B). No significant differences in DRD2 membrane level were observed in the different experimental conditions by confocal analysis (Fig 3A) or by BPA labelling (Fig 3C).

**Fig 4:**
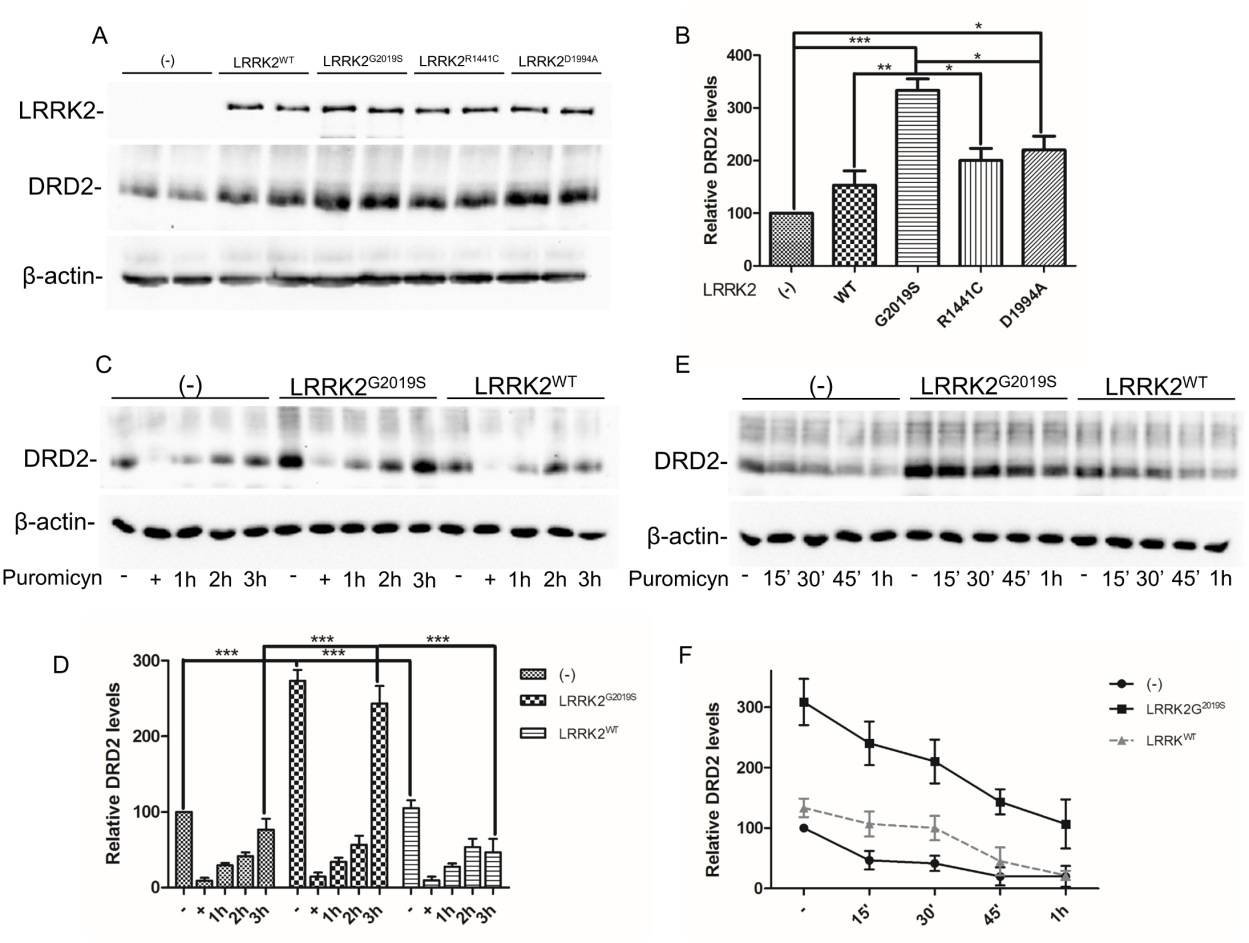
Analysis of LRRK2 effect on DRD2 total protein level. **(A)** SH-SY5Y cells stably expressing FLAG-tagged DRD2 were transduced by the different LRRK2 isoforms. 48h after transduction, protein extracts were prepared and subjected to SDS-PAGE and western blot. The indicated proteins were visualized by western blot using specific antibody. β-actin serves as controls for equal loading of samples. **(B)** Quantification of data obtained in (A). The data represent the mean ± SD. *p<0,05; **p<0,01; ***p<0,001. One-way ANOVA and Bonferroni post test were used. **(C)** SH-SY5Y-DRD2 transduced as in (A) were treated for 2 hours by puromycin (+), then the compound was removed and the new DRD2 protein synthesis was analyzed at 1h, 2h and 3h. Cell lysates were prepared and analysed by western blot. **(D)** Relative band densitometry of data obtained in (C) normalized by the untransduced cells. The data represent the mean ± SD. ***p<0,001 Two-way ANOVA and Bonferroni post test were used. **(E)** SH-SY5Y-DRD2 transduced as in (A) were treated for different time points (15', 30', 45', 60') by puromycin, then cell lysates were prepared and analyzed by western blot. DRD2 decrease was visualized by specific anti-FLAG antibody. **(F)** Relative band densitometry of data obtained in (D) normalized by the untransduced cells. The data represent the mean ± SD.

We reasoned that a change in DRD2 localization may result in alteration in DRD2 total protein level in the presence of LRRK2 over-expression. Stable SH-SY5Y-DRD2 cells were transduced by the different LRRK2 isoforms and the total DRD2 protein level was analyzed 48 hours after transduction. As shown in Fig 4A and 4B, the over-expression of LRRK2 mutants determines a slight but significant increase in DRD2 total protein level compared to LRRK2 WT and an important increase compared to untransduced cells. In the same experimental conditions, no significant difference in DRD1 total levels was detectable in the presence of any LRRK2 transgenes as shown in Fig 2A. We deeply analysed whether the DRD2 increased protein level may be due to a higher transcription rate or an increase in protein synthesis. Therefore, we have analyzed the DRD2 mRNA level by real-time PCR in the presence or absence of G2019S LRRK2. No difference in DRD2 mRNA level was detected 48 hours after adenoviral transduction compared to untransduced cells (S3C Fig). We then compared *de novo* protein synthesis after short (two hours) protein translation block by puromycin. As shown in Fig 4C and 4D, the level of DRD2 protein synthesis was comparable between the different experimental samples at early time points (1 or 2 hours), strongly indicating that LRRK2 expression does not alter DRD2 protein translation rate. However, at 3 hours we can detect significant differences in DRD2 level in the presence of G2019S LRRK2 that likely reflects an alteration in localization and/or degradative pathways. No differences in beta-actin protein levels were detected by this short puromycin treatment. Then, we have evaluated the DRD2 turnover by blocking protein synthesis, by puromycin treatment, 48 hours after transduction of SHSY5Y-DRD2 cells by WT or G2019S LRRK2 recombinant adenovirus. As shown in Fig 4E and 4F, the degradation rate slower in G2019S LRRK2 cells than in untransduced cells. Roughly 50% of DRD2 is degraded in untransduced cells after 15 minutes of puromycin treatment compared to the roughly 20% decrease in cells transduced by G2019S LRRK2. Cells transduced by LRRK2 WT show an intermediate phenotype.

Taken together, these data indicate that LRRK2 pathological mutants may slow down the DRD2 trafficking between the Golgi and the plasma membrane and likely its half-life, since the fraction of DRD2 reaching the cell membrane is degraded upon internalization. Our results do not exclude a direct involvement of LRRK2 in the autophagic-lysosomal pathways that in turn may affect the Golgi physiological function.

### LRRK2 expression alters both DRD1 and DRD2 signalling

To follow the DR activation pathways we assayed the phosphorylation of Extracellular signal Regulated Kinases 1 and 2 (ERK1 and ERK2). In fact, results obtained from different cell culture systems suggest that both D1- and D2-class dopamine receptors can regulate ERK phosphorylation/activation [30–33].

**Fig 5:**
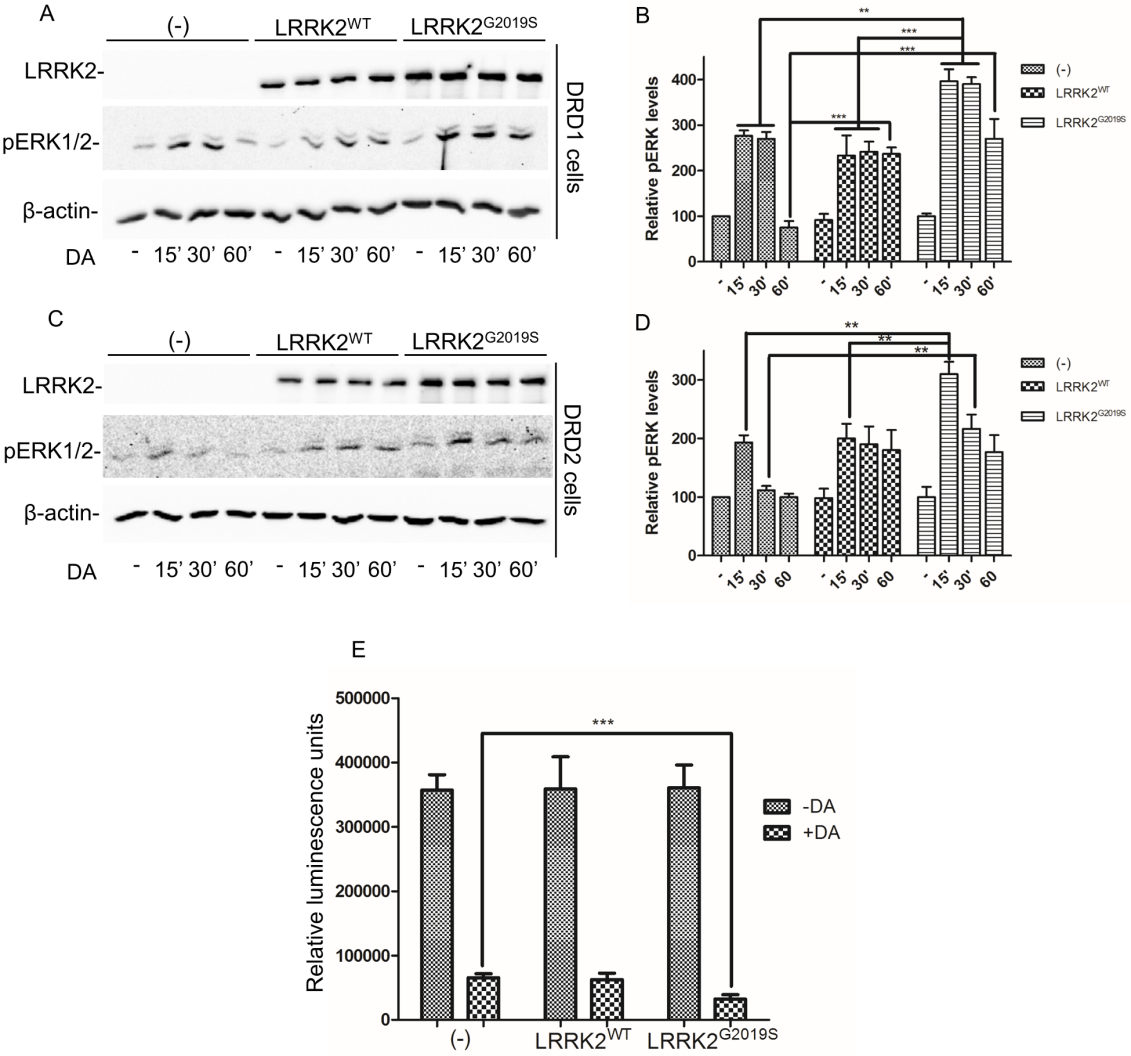
Analysis of DRD1 and DRD2 signalling upon dopamine treatment in SH-SY5Y-DRD1 or -DRD2 stable lines untransduced or transduced by WT or G2019S LRRK2. **(A)** Cells stably expressing FLAG-tagged DRD1 were transduced by the different LRRK2 isoforms. 48h after transduction the cell were treated for different time points (15', 30', 60') by dopamine. Cell lysates subjected to western blot using specific antibody for the indicated proteins. β-actin serves as controls for equal loading of samples. **(B)** Relative band densitometry for pERK of data obtained in (A) normalized by untransduced and untreated cells. The data represent the mean ± SD. **p<0,01; ***p<0,001. Two-way ANOVA and Bonferroni post test were used. **(C)** Cells stably expressing FLAG-tagged DRD2 were treated as before and analyzed by western blot. **(D)** Relative band densitometry for pERK of data obtained in (C) normalized by untransduced and untreated cells. The data represent the mean ± SD. **p<0,01. Two-way ANOVA and Bonferroni post test were used. **(E)** The SH-SY5Y-DRD1 were treated as before and analyzed for cAMP level at basal level (-DA) or upon 15 minutes of dopamine treatment (+DA). The assay is measuring the ATP decrease due cAMP generation. ***p<0,001. One-way ANOVA and Bonferroni post test were used.

SH-SY5Y-DRD1- or -DRD2 cells were transduced by recombinant adenovirus expressing WT or G2019S LRRK2. Fourty-eight hours after transduction, cells were kept for 1 hour without serum and dopamine was added at different times (15, 30 or 60 minutes). Protein extracts were then analyzed by western blot using a specific antibody against phospho-ERK. As shown in Fig 5A and 5B, the presence of G2019S LRRK2 determines a strong ERK activation upon DA treatment in the SH-SY5Y-DRD1 cells and this activation is more persistent than in control untransduced cells, with the cells transduced by WT LRRK2 showing an intermediate phenotype. Comparable effect on ERK phosphorylation was observed in SH-SY5Y cells expressing DRD2 and transduced by the different LRRK2 isoforms upon dopamine stimulation (Fig 5C and 5D). These results suggest that the over-expression of WT and G2019S LRRK2 modifies the DRD1 and DRD2 signalling, most probably through an alteration of receptor trafficking. To further analyze the DRD1 signalling in the presence of LRRK2, we measured cAMP generation upon dopamine treatment in SH-SY5Y-DRD1. As shown in Fig 5E, DA exposure of SH-SY5Y-DRD1 transduced by G2019S LRRK2 determines a stronger and significant decrease of ATP level (that indicates an increase in cAMP generation).

### Analysis of DRD1 trafficking in LRRK2 animal models

We first tested the most suitable commercial anti-DRD1 or anti-DRD2 antibodies to detect endogenous proteins in mouse brain and primary neurons. The commercial anti-DRD1 antibody (Sigma D2944) is able to detect the DRD1 protein either by western blot (without boiling the protein samples) or by immunofluorescence. Unfortunately, under our experimental conditions, no commercial anti-DRD2 antibodies were able to detect the receptor either by western blot or immunofluorescence. As a consequence we have analyzed the tissues and primary neurons only for DRD1 expression.

**Fig 6:**
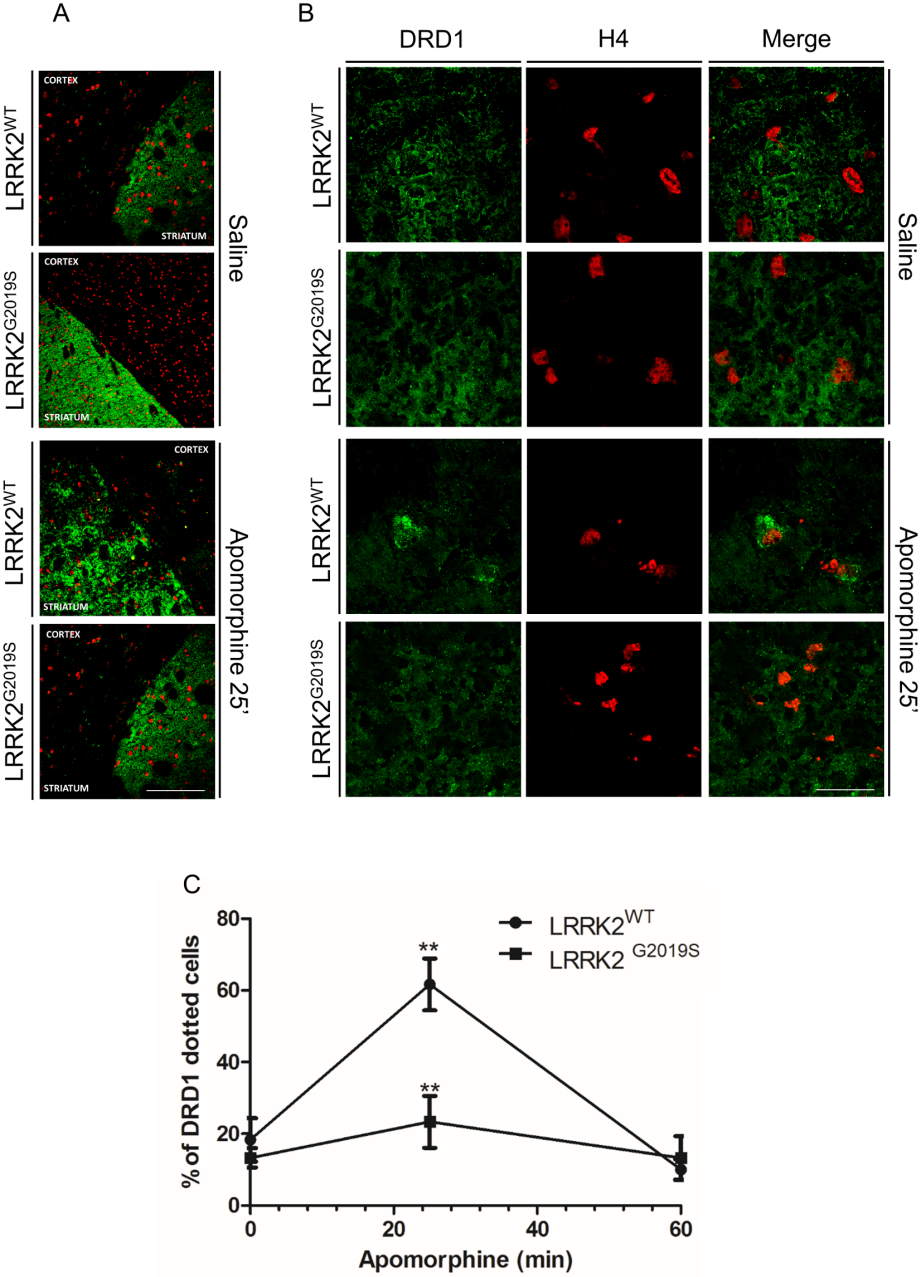
Analysis of DRD1 internalization in G2019S LRRK2 knock-in mice. **(A and B)** DRD1 localization/internalization at low (A) and high magnification (B) upon 25 minutes of saline (saline) or apomorphine treatment (apomorphine) of G2019S LRRK2 knock-in mice or WT. After agonist treatment the mouse brain were quickly dissected and frozen in embedding medium. Cryostat sections were incubated with the different primary antibodies (anti-DRD1 or histone H4) and with Alexa488-conjugated secondary antibody (green) or Alexa647-conjugated secondary antibody (red) for DRD1 or H4 respectively. Scale bars = 250 μm and 25μm for A and B respectively. **(C)** Quantification of data obtained in (B) indicating the percentage of dotted nuclei obtained by the analysis of at least twenty high magnification confocal pictures for each animal. The data represent the mean ± SD. **p<0,01. Two-way ANOVA and Bonferroni post test were used.

We extended our study on DRD1 trafficking to the striatum of 4-month old male mice knock-in for G2019S LRRK2 compared to age-matched WT LRRK2 mice. Western blot analysis showed no difference in DRD1 level in total, membrane or vesicle fractions between WT or G2019S LRRK2 knock-in mice, either in basal conditions or upon treatment with apomorphine (3 mg/Kg) for 25 minutes to stimulate DR trafficking (S3A and S3B Fig). However, immunofluorescence analysis on striatal sections highlighted significant differences between the two genotypes. In particular, 25 minutes of apomorphine treatment resulted in a pronounced dotted DRD1 staining (likely representing vesicle structures) in WT compared to G2019S knock-in mice (Fig 6B and 6C). No significant differences were visible in saline treated animals between the two different genotypes.

**Fig 7:**
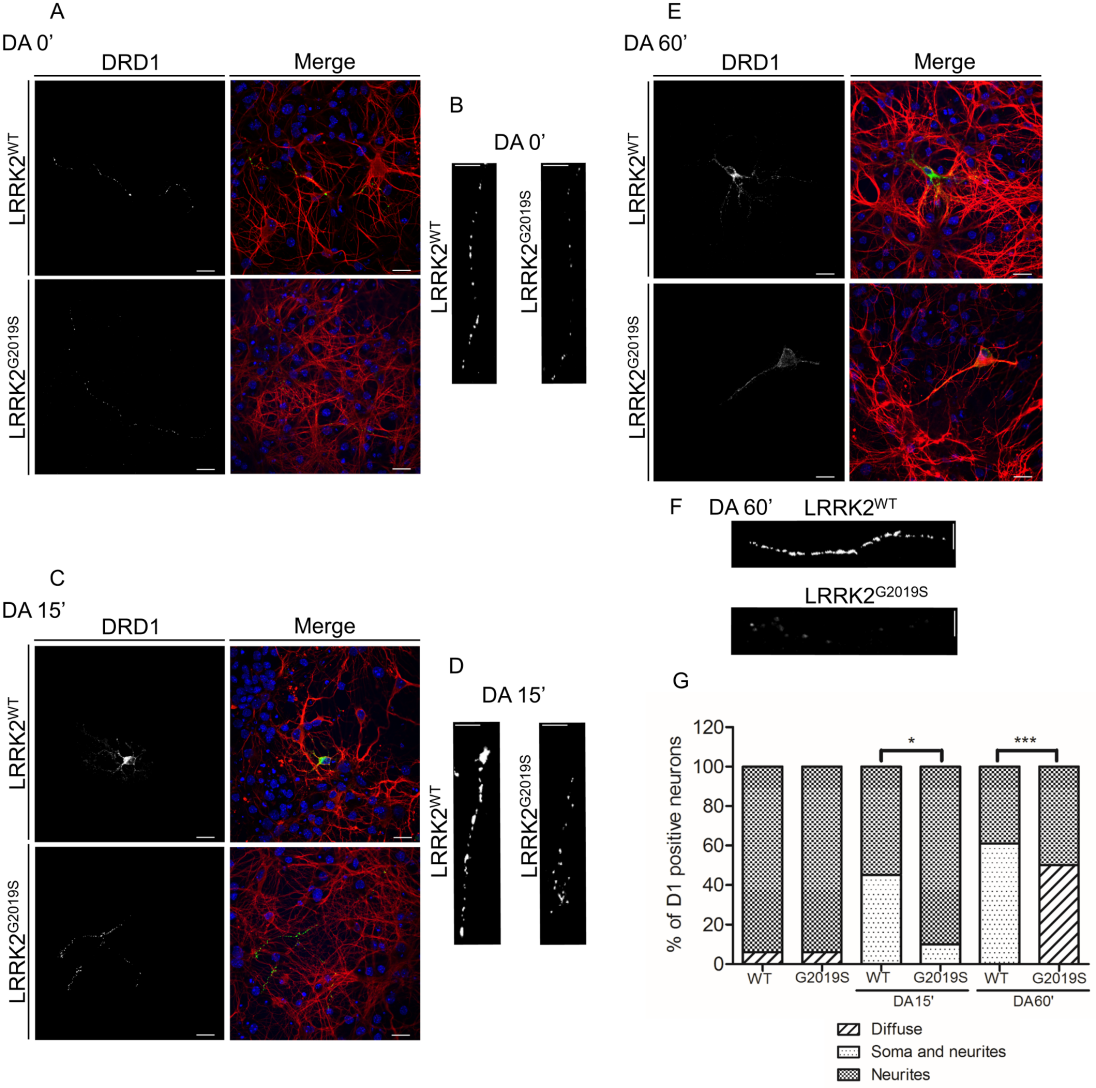
Analysis of DRD1 localization/trafficking in striatal primary neurons from WT and G2019S LRRK2 knock-in. **(A, C and E)** DRD1 trafficking at basal conditions (A) or upon 15 (C) or 60 minutes (E) of dopamine treatment on DIV7 striatal primary neurons isolated from G2019S LRRK2 knock-in mice or WT. After agonist treatment, the cells were fixed and incubated with the different primary antibodies (anti-DRD1 or beta III tubulin) and with Alexa488-conjugated secondary antibody (green) or Alexa647-conjugated secondary antibody (red) for DRD1 or beta tubulin respectively. Scale bars = 20μm **(B, D and F)** High magnification of DRD1 neurite labelling at basal conditions (B) or upon 15 (D) or 60 minutes (F) of dopamine treatment. Scale bars = 10μm. **(G)** Quantification of data obtained in (7A, 7B, 7C, 7D, 7E and 7F). The data represent the percentages of diffuse, somato-neuritic and neuritic D1 puncta distribution in D1 positive neurons from two independent replicates and are represented as mean ± SEM. *p<0,05; ***p<0,001. Two-way ANOVA and Bonferroni post test were used.

To further support these results we analysed the DRD1 trafficking in primary striatal neurons isolated from the same WT or G2019S LRRK2 knock-in mice. In primary neurons DRD1 is mainly detected as small fluorescent puncta along the membrane surface of neurites (Fig 7A) and [34]; upon agonist treatment, the labelling extends to the soma and the neurite puncta appear larger and brighter (Fig 7C and 7E) and [34]. The presence of G2019S LRRK2 determines, at basal level, a significant decrease in fluorescent puncta size (Fig 7A and S2C Fig) in neurites compared to WT primary striatal neurons. Moreover, upon agonist treatment, DRD1 localization/trafficking is significantly altered in the presence of G2019S LRRK2. In particular, at 15 minutes of dopamine treatment, in WT primary neurons a significant soma staining is visible compared to G2019S neurons (roughly 44% in WT vs 10% in G2019S) (Fig 7C and 7G); at 60 minutes, the DRD1 staining is largely diffused in knock-in compared to the brighter DRD1 staining of neurites and soma in WT primary neurons (Fig 7E and 7G).

## Discussion

The catecholaminergic neurotransmitter dopamine acts by binding five specific G protein-coupled dopamine receptors (D1, D2, D3, D4, and D5) and controls fundamental physiological functions ranging from the control of voluntary movement, reward pathways, hormonal regulation and, not least, hypertension. Among them, DRD1 and DRD2 are the most abundant. Both D1 and D2 receptors are highly expressed along the nigrostriatal, mesolimbic, and mesocortical pathways. Transcripts from the Drd2 gene lead to the generation by alternative splicing of two major D2 dopamine receptor variants that have been termed DRD2S (D2-short) and DRD2L (D2-long) [23, 24]. The DRD2 alternative splicing between introns 4 and 5 determines the presence of 29 amino acids in the third intracellular loop that are lacking in the short isoform. DRD2L and DRD2S have distinct anatomical, physiological, signalling and pharmacological properties. D2S has been shown to be expressed at higher level pre-synaptically and to be mostly involved in autoreceptor functions, whereas D2L is predominantly a postsynaptic isoform [22]. Interestingly, DRD2L and DRD2S also show a different cellular distribution in neuroblastoma NG108-15 cells, with DRD2S mainly localized into cell membrane and DRD2L distributed between the Golgi apparatus and the plasma membrane [29]. The dopamine receptor trafficking may have a prominent role in the regulation of dopaminergic neuron physiology and, in fact, many studies show both in animal models of Parkinson's disease, as well as in patients, that the density of D1 and D2 receptors changes although this alteration could be merely in association with the developing supersensitivity due to DA denervation. Interestingly, recently it has been shown an important role of dopamine D1 receptor in the control of the immune systems by negatively regulating the inflammasome [35]. Extensive experimental evidence indicates that inflammatory processes are instrumental in neuronal cell death in PD and LRRK2 seems to have a prominent role in inflammatory cells.

DRD1 rapidly internalizes in response to dopamine and recycles *in vivo* [36, 37], in contrast, DRD2 is significantly down regulated *in vivo* after prolonged drug abuse [38], in animals with increased dopamine tone due to deletion of the dopamine transporter [39], or after chronic low doses of D2R agonists [40]. These data parallel those obtained in the majority of experiments using cultured cells stably expressing DRD1 or DRD2 [25], although with some discrepancies for DRD2 [28]. Our SH-SY5Y cell lines largely recapitulate the dopamine receptor trafficking observed *in vivo* and *in vitro* in most of experimental models. In these cells, LRRK2 plays an important role in the control of DR trafficking likely by a modulation of vesicle trafficking. In SH-SY5Y cells stably expressing the DRD1, expression of G2019S LRRK2 determines a strong impairment in DRD1 internalization upon dopamine treatment. This effect is not visible in the presence of either WT or A53T synuclein, excluding any possible artefact in our assay due to recombinant protein expression or to adenovirus transduction. The reduced internalization of DRD1 in the presence of G2019S LRRK2, even at short time treatment (5 minutes), strongly suggests an alteration in endocytosis more than an increase in recycling/exocytosis pathway. Importantly, these results extensively mirror the alteration in DRD1 trafficking observed in primary knock-in neurons expressing the G2019S LRRK2 at endogenous level. In particular, wild type primary neurons exhibit extensive DRD1 positive clusters of neuronal soma upon dopamine treatment that is significantly reduced in knock-in neurons. Moreover, the G2019S expression *per se* determines a significant alteration in the size of DRD1 puncta staining in neurites, strongly suggesting an important effect of G2019S pathological mutant on DRD1 localization/clustering.

A DRD1 clustering/internalization impairment, upon apomorphine treatment, is detected also on brain sections, by immunofluorescence analysis, in G2019S LRRK2 knock-in mice compared to WT mice. Unfortunately, we were not able to confirm these data by DRD1 western blot analysis on striatal protein extracts of these animals, but this discrepancy could be explained by the complexity of the striatal tissue; for instance, striatal neurons differ in terms of firing rate and timing in response to agonist stimulation.

The internalization impairment is likely independent from DRD1 phosphorylation, since the receptor band undergoes a similar molecular weight shift when analysed by SDS-PAGE in the absence or presence of WT or G2029S LRRK2. The DRD1 trafficking alteration in the presence of G2019S LRRK2 parallels a change in its signalling, with a higher cAMP generation and ERK phosphorylation, strongly suggesting that the impairment in receptor internalization may alter signalling transduction shutdown. Taken together, our results indicate that G2019S LRRK2 is altering some molecular mechanism that mediates the internalization of DRD1 following the receptor phosphorylation due to agonist treatment but also the basal distribution of the receptor in unstimulated conditions.

An alteration in receptor trafficking is detectable also in our SH-SY5Y-DRD2 stable line. All LRRK2 pathological mutants lead to a significant accumulation of DRD2 into the Golgi apparatus compared to WT LRRK2 or untransduced cells. These results are of particular interest since all LRRK2 pathological mutants show a similar phenotype suggesting the possibility of a common or converging pathological mechanism. The DRD2 accumulation into the Golgi correlates with a significant increase of DRD2 total protein levels. The presence of any LRRK2 variant does not significantly alter the DRD2 transcriptional or translational rate. Conversely, expression of mutant LRRK2s determines a slight but significant decrease in DRD2 degradation (compared to untransduced or WT LRRK2) likely due to the DRD2 accumulation into the Golgi. Interestingly, the membrane level of DRD2 in the presence of WT or G2019S LRRK2 are quite similar despite significant differences in total protein level, suggesting that either a small amount of DRD2 is slowly accumulating into the Golgi or that other compensatory mechanisms take place on the cell membrane. A possible explanation might be that physiologically a certain amount of DRD2 is kept into the Golgi apparatus (and maybe also in the outpost Golgi in neurons) and that mutant LRRK2s affect its vesicle-mediated transport to the cell membrane. We can speculate that LRRK2 is part of a protein complex that negatively regulates the Golgi-membrane trafficking. Over-expression of WT LRRK2 and even more the pathological mutant is likely increasing the formation/activity of this complex, that may partially lock the DRD2 into the Golgi apparatus. A different hypothesis could be a more general alteration in Golgi apparatus, for instance LRRK2 seems to affect Golgi clearance by autophagy [10]. As previously mentioned, different LRRK2 interactors belong to protein families involved in the regulation of vesicle trafficking [7, 9–13] or involved in the regulation of the cytoskeleton dynamics that in turn may modulate vesicle trafficking [14–17]. Any of these LRRK2 interactions may be responsible for DRD1 or DRD2 trafficking modulation that we observe in the presence of LRRK2. Our data correlate, to a different extent, to the alteration of DR level or signalling observed in LRRK2 mouse models. In particular, the PKA-dependent phosphorylation of GluR1 is aberrantly enhanced in the striatum of young and aged LRRK2-null mice after treatment with a DRD1 agonist [14] and the total DRD2 protein level is higher in mice over-expressing WT LRRK2 [18]. Moreover, our results correlate with accumulating evidences that demonstrate a critical role of LRRK2 in the vesicle trafficking machinery that transports proteins from dendritic ER exit sites to Golgi outpost and to dendritic surface [11]. In particular, it has been reported that either LRRK2 loss or R1441C missense mutation impair the activity-dependent trafficking of NMDRs in neurons [11].

**Fig 8:**
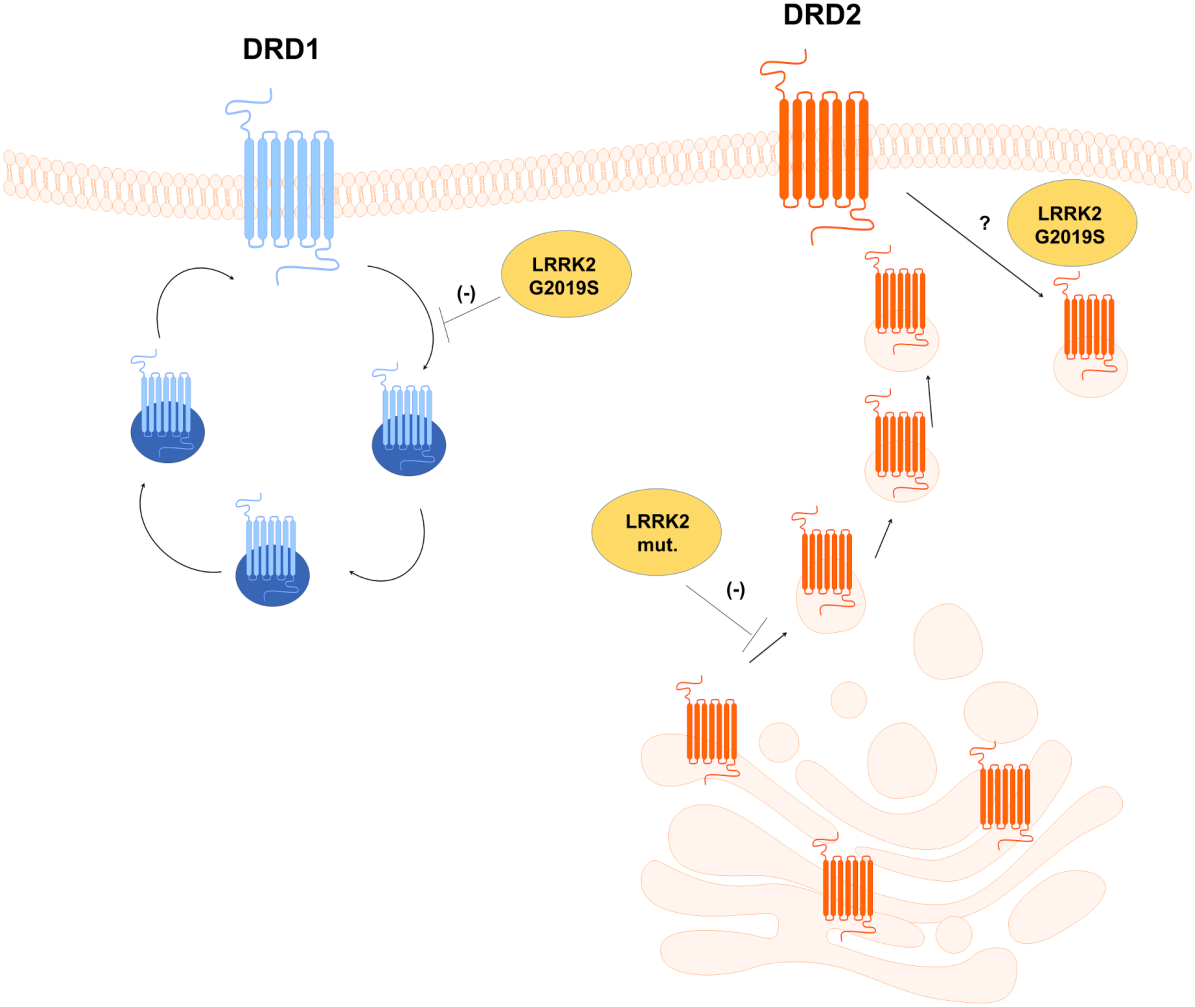
Model for mutant LRRK2 action in the alteration of dopamine receptor trafficking. The LRRK2 pathological mutant G2019S and R1441C alter the DRD2 protein localization likely affecting the DRD2 trafficking form the Golgi to cell membrane. Moreover the mutant G2019S is negatively regulating the DRD1 internalization upon dopamine stimulation. For both dopamine receptors expression of the mutant LRRK2 alter signalling pathways upon dopamine stimulation.

Fig 8 summarizes a general view that arises from our and other published results on the involvement of LRRK2 in the regulation of dopamine receptor trafficking. Mutant LRRK2s modulate the DRD2 protein level likely affecting the trafficking from Golgi to cell membrane. The intacellular DRD2 is locked into the trans Golgi as indicated by the significant overlap with the TGN46 marker. Interestingly, this effect is common to all LRRK2 pathological mutants. More complex is the involvement of LRRK2 in the control of DRD1 trafficking. Only G2019S LRRK2 impairs DRD1 internalization upon dopamine treatment. We can speculate about some specific pathways altered by G2019S LRRK2 mutant likely related to its hyperactive kinase function compared to other LRRK2 pathological mutants, although this view is slight challenged by the recent discovery that the majority of LRRK2 mutations in the cellular context, but not *in vitro*, exhibit increased phosphorylation of its possible substrates [12].

Further experiments are required to better understand the molecular mechanisms of LRRK2 regulation of dopamine receptor trafficking and to determine whether this effect is specific for dopaminergic receptors or it is a general mechanism regulating different neuronal membrane receptors.

## Materials and methods

### Animals

Male homozygous LRRK2 G2019S KI mice backcrossed on a C57Bl/6J background were raised at the University of Ferrara [41, 42]. Male non-transgenic wild-type (WT) mice were littermates obtained from the respective heterozygous breeding. Mice employed in the study were kept under regular lighting conditions (12 h light/dark cycle) and given food and water *ad libitum*. Experimental procedures involving the use of animals were approved by the Italian Ministry of Health (license 318/2013-B). Mice were killed by rapid cervical dislocation, adequate measures were taken to minimize animal pain and discomfort.

### Reagents and solutions

Tween^®^ 20 (Polyethylene glycol sorbitan monolaurate), protease inhibitor cocktail, Dopamine hydrochloride, IGEPAL^®^ CA-630 (Octylphenoxy poly(ethyleneoxy)ethanol), L-Glutathione reduced, Streptavidin−Agarose from Streptomyces avidinii, Collagen type IV, IBMX (3-Isobutyl-1-methylxanthine), Ro 20–1724 (4-(3-Butoxy-4-methoxybenzyl)imidazolidin-2-one), R-(−)-Apomorphine hydrochloride hemihydrate were obtained from Sigma-Aldrich (Milano, Italy). EZ-Link^™^ Sulfo-NHS-Biotin was from Thermo Scientific. LRRK2 inhibitors: CZC-25146 and HG10-102-01 from Calbiochem. The phosphate-buffered saline (PBS) solution was made using NaCl (137 mM), KCl (2.7 mM), Na2HPO4 (8.1 mM), KH2PO4 (1.47 mM) from Sigma and then adjusted to pH 7.4. Dulbecco’s modified Eagle’s medium (DMEM)–F12, Fetal Bovine Serum (FBS), Streptomycin/Penicillin, Geneticin-G418 were purchased from Life Technologies.

### Plasmid constructions

cDNA corresponding to human LRRK2 (accession no. NM_198578) was RT—PCR amplified from human lymphoblast mRNA and cloned as previously described in [43]. *PacI* recognition site on LRRK2 cDNA was mutagenized by site-directed mutagenesis (forward TTGAGAAATTAATCAAACAGTGTTTG, reverse CAAACACTGTTTGATTAATTTCTCAA) without changing the amino acidic sequence. pShuttle2-LRRK2 (WT or R1441C or D1994A or G2019S) were obtained by digestions of cDNAs corresponding to human LRRK2 without *PacI* restriction site with *NotI* and *XbaI* and subcloned in *DraI/XbaI* cloning sites in pShuttle2 vector (Clontech). Expression cassettes containing LRRK2 cDNAs were excised from pShuttle2 and subcloned into pAdeno-X vector according to Adenoviral-X Expression System 1 (Clontech). Plasmids to generate DRD1 or DRD2 stable cell lines were obtained as previously described in [20].

### Adenoviral delivery

Adenoviral particles were produced and titrated using the Adenoviral-X Expression System 1 (Clontech) according to manufacturer’s instruction. Cells were transduced by adenoviral particles (10–30 pfu/cell) in DMEM-F12 and incubated at 37°C for 1 h. The transduced cells (usually more than 90% expressing LRRK2) were analyzed 48h transduction.

### Cell lines and SH-SY5Y stable clones

Human neuroblastoma SH-SY5Y cells (ATCC number CRL-2266) were grown in DMEM–F12, 10% Fetal Bovine Serum (FBS) at 37°C. The plasmid pcDNA3.1 containing cDNAs coding for DRD1-Flag or DRD2-Flag were transfected using Lipofectamine^®^ LTX Reagent (Life Technologies) according to the manufacturer’s protocol. The different SH-SY5Y clones were maintained under selection by 400 μg/mL of G418. Individual clones were picked after 14 days of selection, moved in a 96 well plate, and maintained under selective medium until confluence growth. Different individual clones were analyzed for DRD1 or DRD2 expression by western blot and immunofluorescence.

### Biotin protection/degradation assay (BPA)

1x10^6^ SH-SY5Y-DRD1 or SH-SY5Y-DRD2 cells were grown in a collagen type IV (Sigma-Aldrich) coated 6-well plates. After 24 hours, cells were transduced by recombinant adenovirus and 48 later subjected to the biotin protection assay protocol as described in [25]. Briefly, cells were treated with 0.3 mg/ml Sulfo-NHS-SS-Biotin for 30 min at 4°C. Cells were then washed in PBS 1X 1 mM MgCl_2_, 0.1 mM CaCl_2_, pH 8.2 and placed in DMEM–F12 pre-warmed medium for 15 min before treatment with 10μM Dopamine hydrochloride (or no treatment). After ligand treatment, plates were washed in PBS 1X containing 1 mM MgCl_2_, 0.1 mM CaCl_2_, pH 8.2, and remaining cell surface-biotinylated receptors were stripped in 75 mM NaCl, 1 mM MgCl_2_, 0.1 mM CaCl_2_, 50 mM reduced glutathione (GSH), 80 mM NaOH, 10% FBS pH 8.6 at 4°C for 30 min. Cells were then washed in PBS 1X containing 1 mM MgCl_2_, 0.1 mM CaCl_2_, pH 8.2 and lysed by 150 mM NaCl, 1% NP40, 20mM Tris-HCl pH 7.5, protease inhibitor cocktail. Cellular debris were removed by centrifugation at 13,000xg. Cleared lysates were precipitated by Streptavidin-Agarose beads overnight at 4°C. Beads were washed 4 times with 20 mM, 150 mM NaCl, 1% IGEPAL, 20mM Tris-HCl pH 7.5. Samples were then resolved by SDS-PAGE.

### Subcellular fractionation of cells or mouse tissues

Tissues from 4 months old LRRK2WT or LRRK2G2019S KI male mice were quickly dissected and frozen. Subcellular fractionation was conducted as described in (40). Briefly, striatum were homogenized in ice-cold homogenization-buffer (320 mM sucrose, 4 mM HEPES, pH 7.4, protease inhibitor cocktail (Sigma)). The homogenates were centrifuged at 1000xg for 10 min to produce the pellet containing nuclei and large debris fraction (P1). The supernatant (S1) was further fractionated into pellet (P2 containing the membrane fraction) and supernatant (S2) by centrifugation at 10,000xg for 20 min. The S2 was ultracentrifuged at 100,000xg g to obtain the pellet (P3 containing the vesicle fraction). Protein content was determined using the Bradford protein assay. Equal amount of protein extracts were loaded into the SDS-PAGE.

### Western blot analysis

Western blot analysis was performed as previously described [20]. Briefly, protein content was determined using the Bradford protein assay. Equal amounts of protein extracts were resolved by standard sodium dodecyl sulfate-polyacrylamide gel electrophoresis. Samples were electroblotted onto Protan nitrocellulose (GE Healthcare Life Sciences). Membranes were incubated with 3% low-fat milk in 1X PBS-Tween 0.05% solution with the indicated antibody: anti-LRRK2 (1:5000 MJFF2 c41-2 Epitomics), anti-Flag (F3165 1:2500 Sigma-Aldrich), anti-DRD1 (D2944 1:2000 Sigma-Aldrich), anti-beta-actin (A5441 1:5000 Sigma-Aldrich), Phospho-p44/42 MAPK (Thr202/Tyr204) (9101 1:1000 Cell Signalling) for 16 h at 4°C. Goat anti-mouse immunoglobulin G (IgG) peroxidase-conjugated antibody (1:2500 Millipore Corporation) or goat anti-rabbit IgG peroxidase-conjugated antibody (1:5000 Millipore Corporation) were used to reveal immunocomplexes by enhanced chemiluminescence (Pierce Biotechnology, Rockford, IL, USA).

### Primary striatal cultures

Primary midbrain cells were prepared from postnatal C57black/J6 P0 mice brains. Briefly, tissues from 8–9 pups were dissociated with 199 units of Papain and 0,1% DNase (Worthington Biochemical Corporation) in 5ml of Earle's Balanced Salt Solution (EBSS) for 30 minutes at 37°C, followed by trituration. Enzymatic digestion was stopped with EBSS containing papain inhibitor. Intact cells were separated from cell debris by centrifugation through a single step of discontinuous density gradient in presence of 10mg/ml serum bovine albumin and 10mg/ml ovomucoid in EBSS. Cellular pellet was then resuspended in Neurobasal media (Life Technologies) supplemented with 2% v/v of B27 (Invitrogen), 0.5 mM L-glutamine (Life Technologies), penicillin (100 units/ml) streptomycin (100 μg/ml) and 2.5 μg/ml fungizone (Life Technologies). Cells were then plated at a density of 2 × 10^5^ cells/well on poly-L- lysine-coated wells (0.1 mg/ml, Sigma Aldrich) in a 24-well plate and cultured at 37 °C in 5% CO_2_. At DIV7 neurons were treated with 10μM dopamine for 15 or 60 minutes, fixed in 4% paraformaldehyde and then processed for immunofluorescence. Fluorescent images of D1 positive neurons were acquired using a Zeiss LSM 700 confocal microscope equipped with a 63× oil objective. 8–10 images/replicate/condition were taken from a single z-plane at a thickness of 2,2 μm and exposure settings for the 488 fluorochrome were kept constant across images and experiments. Neuronal images were analyzed by using the Analyze particle macro of ImageJ and the area of D1 fluorescent puncta was measured. Two independent primary culture preparations (from 8 pups each) were used per genotype and per condition. Images of twenty DRD1 positive neurons were taken for each experimental point per genotype. In all neuronal pictures, D1 puncta distribution was scored as diffuse, neuritic or somato-neuritic and reported as percentage of total D1 positive neurons.

### Immunofluorescence

For cells: 1×10^5^ SH-SY5Y-DRD1 or SH-SY5Y-DRD2 cells, grown on a cover-glass, were washed twice with PBS 1X and then fixed with 4% paraformaldehyde/PBS for 10 min. Cells were permeabilized with 0.1% Triton X-100 diluted in PBS. For tissue: after apomorphine hydrochloride (3mg/Kg) treatment the mice were sacrificed. The brains were embedded in OCT freezing medium and 10 μm-thick sections were prepared by cryostat. All sections were fixed with 4% paraformaldehyde/PBS for 15 min and washed with 0.05% Tween-20 diluted in PBS. Non-specific binding was blocked with 5% bovine serum albumin, 0.05% Tween-20 diluted in PBS for 1 h at room temperature. Cells or tissue were incubated with primary antibodies: anti-LRRK2 (1:500 Mjff2 c41-2 epitomics), anti-Flag (1:2500 Sigma-Aldrich), anti-alpha-synuclein (1:500, Millipore), anti-DRD1 (1:1000, Sigma-Aldrich), anti-H4 histone (1:5000, Sigma-Aldrich), anti-TGN46 (MBS223409, 1:1000 Mybiosource), mouse anti-beta III tubulin (clone 2G10, 1:500 Sigma) diluted in blocking solution, overnight at 4°C. Cells were then washed with PBS, 0.05% Tween-20 diluted in PBS and incubated with secondary antibodies: Goat anti-Mouse IgG Secondary Antibody Alexa Fluor^®^ 488 (Life Technologies) and Goat anti-Mouse IgG Secondary Antibody Alexa Fluor^®^ 647 (Life Technologies) diluted 1:1000 in blocking solution for 1 hour at room temperature. Golgi staining was performed with Wheat Germ Agglutinin Alexa Fluor^®^ 633 Conjugate (Life Technologies). Before analysis, cells were mounted using Mowiol mounting medium and fluorescence was revealed with a Leica TCS SP5 confocal microscope with LAS lite 170 image software. For the statistical analysis, four independent animals and at least 10 different confocal pictures (high magnification) for each animal were analysed and scored for any experimental point. For DRD1 localization on tissue sections, "dotted cells" indicate cells with marked green spots around the nucleus.

### Intracellular cAMP determination

SH-SY5Y-DRD1 cells were incubated in sterile 96-well plate with a seeding density of 1x10^4^ cells per well. Cells were cultured in DMEM-F12 10% FBS during 24 hours and transduced by adenovirus encoding LRRK2s for 48 hours. Medium was replaced by serum-free medium containing 10μM Dopamine hydrochloride, 500μM IBMX and 100μM Ro 20–1724 and incubated for different times. Intracellular cAMP level measurement was performed using cAMP-Glo assay kit according to manufacturer’s instruction (Promega Corporation). Luminescence was measured using the Victor^™^ X5 Multilabel Plate Reader (PerkinElmer)

### Quantitative real time RT-PCR

Quantitative real-time PCR was performed on the Qiagen Rotor-Gene^®^ Q, using the BioRad iTaq^™^ Universal SYBR^®^ Green Supermix kit. Triplicate PCR reactions were performed following the manufacturer's recommended amplification conditions. For all the analyses, the amplification of GADPH transcripts has been used as reference for normalization. The final PCR products were quantified using *ΔΔCt* method. The following primer pairs were used:

LRRK2 (fw GATTTCACCATTCAGAAACTC and rv CATGACATTTTTAAGGCTTCC)

GADPH (fw TCACCATCTTCCAGGAGCGAG and rv ACAGCCTTGGCAGCACCAGT)

### Statistical analysis

The results are presented as means ± S.D. or S.E.M. of at least n≥3 independent experiments. Two independent experiment were performed for in vivo analysis (primary cultures and transgenic animals). Statistical evaluation was conducted by one-way ANOVA (for comparisons between genotypes at a single time point) or two-way ANOVA (for comparisons between genotypes over time) and Bonferroni post test. Values significantly different from the relative control are indicated with an asterisk. *p<0,05; **p<0,01; ***p<0,001.

## Supporting information

S1 Fig Analysis of DRD1 internalization upon dopamine treatment in SH-SY5Y-DRD1 cells untransduced or transduced by WT or mutant G2019S LRRK2. **(A and B)** DRD1 localization upon 5 (A) or 60 minutes (B) of dopamine treatment of SH-SY5Y-DRD1 cells transduced by the different recombinant adenovirus for 48h. After agonist treatment, the cells were fixed and incubated with the appropriate primary antibodies (anti-FLAG for DRD1 and anti-LRRK2 (MJFF2) for LRRK2) and with Alexa647-conjugated secondary antibody (red) or Alexa488-conjugated secondary antibody (green) for DRD1 or LRRK2 respectively. Scale bars = 10μm.
(TIF)

S2 Fig **(A and B)** DRD1 localization at basal level (A) or upon 15 minutes (B) of dopamine treatment of SH-SY5Y-DRD1 as in Fig 1 with or without 4 hours of CZC25126 pre-treatment. After agonist treatment, the cells were fixed and incubated by the different primary antibodies (anti-FLAG for DRD1 and anti-LRRK2 (MJFF2) for LRRK2) and with Alexa647-conjugated secondary antibody (red) or Alexa488-conjugated secondary antibody (green) for DRD1 or LRRK2 respectively. Scale bars = 10μm. **(C)** Quantification of the average (mean ± SEM) of D1 puncta area from each image from two independent experiments showed in Fig 7B, 7D and 7E. **p<0,01; ***p<0,001. Two-way ANOVA and Bonferroni post test were used.
(TIF)

S3 Fig **(A and B)** Analysis by western blot of DRD1 protein level on total, membrane or vesicle protein fraction purified from striatum of WT or G2019S knock-in mice treated (B) or not (A) by apomorphine. **(C)** Analysis of DRD2 mRNA by real time PCR in the presence of WT or G2019S LRRK2 mutant in the same experimental conditions of Fig 4.
(TIF)
