# Supplementary figures and images for: Role of LRRK2 in the regulation of dopamine receptor trafficking
Source: PLoS One. 2017 Jun 5;12(6):e0179082. doi: 10.1371/journal.pone.0179082 (PMC5459500; doi:10.1371/journal.pone.0179082)

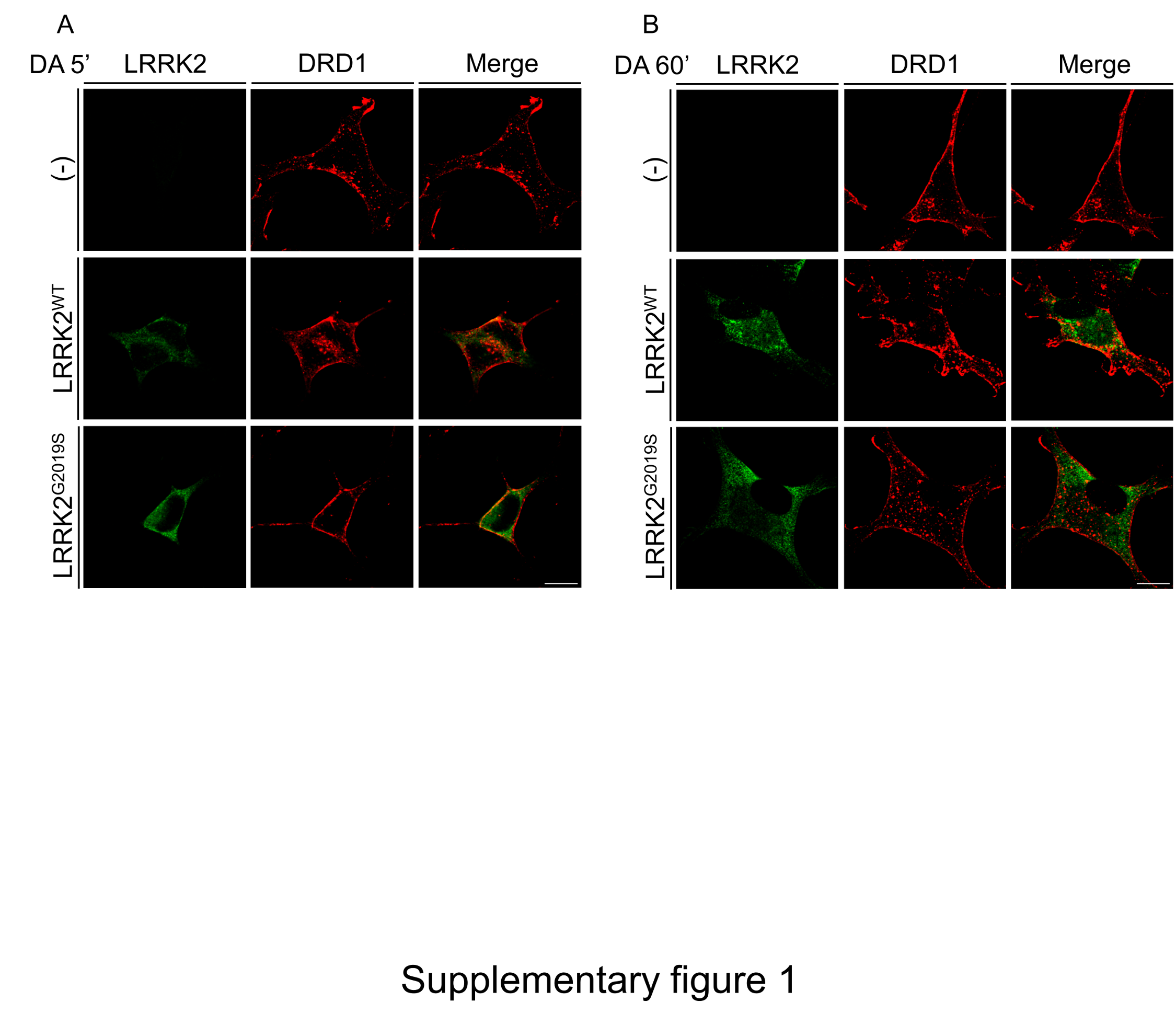

Supplement: S1 Fig — (A and B) DRD1 localization upon 5 (A) or 60 minutes (B) of dopamine treatment of SH-SY5Y-DRD1 cells transduced by the different recombinant adenovirus for 48h. After agonist treatment, the cells were fixed and incubated with the appropriate primary antibodies (anti-FLAG for DRD1 and anti-LRRK2 (MJFF2) for LRRK2) and with Alexa647-conjugated secondary antibody (red) or Alexa488-conjugated secondary antibody (green) for DRD1 or LRRK2 respectively. Scale bars = 10μm. (TIF) [file pone.0179082.s001.tif]

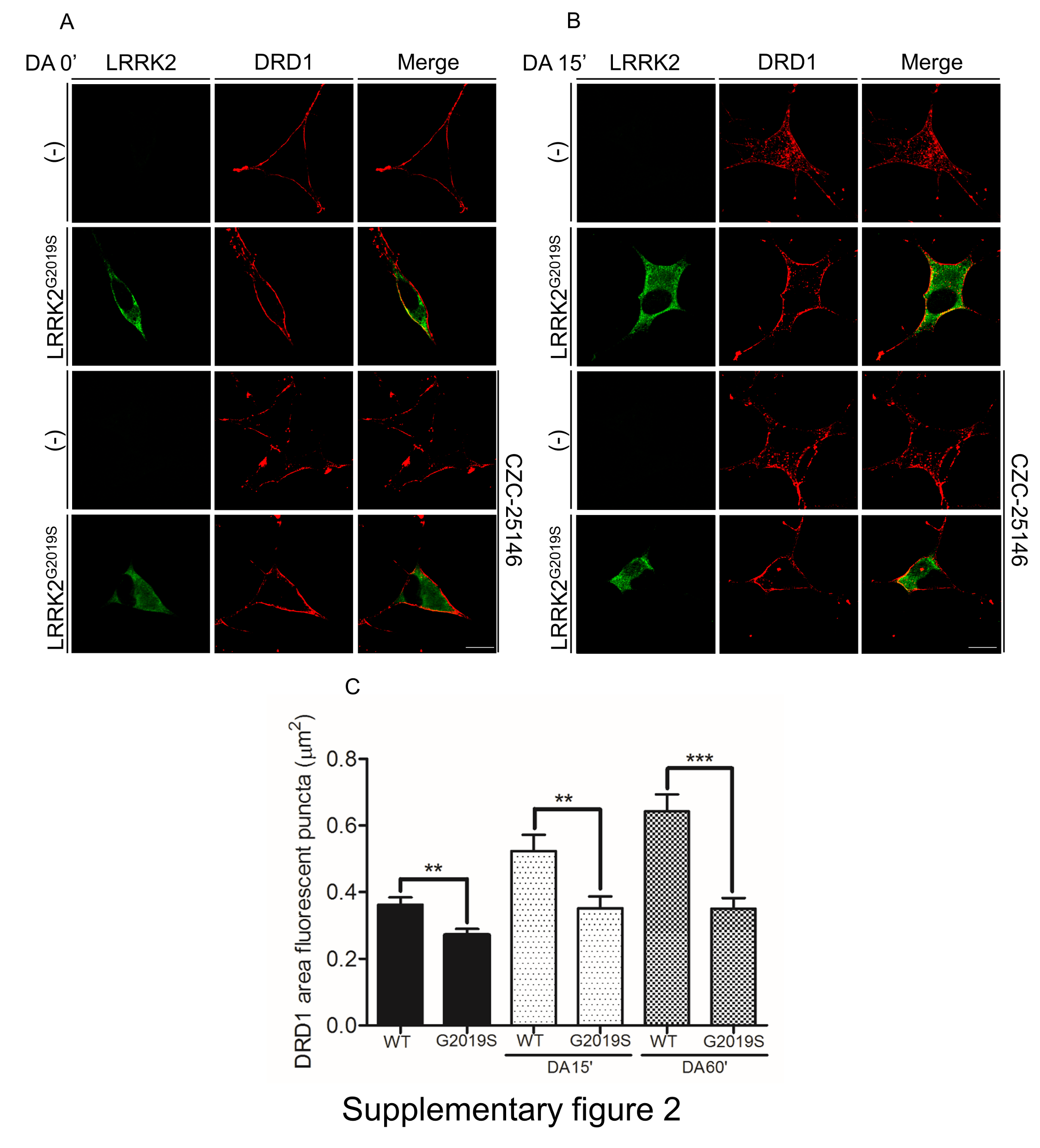

Supplement: S2 Fig — (A and B) DRD1 localization at basal level (A) or upon 15 minutes (B) of dopamine treatment of SH-SY5Y-DRD1 as in Fig 1 with or without 4 hours of CZC25126 pre-treatment. After agonist treatment, the cells were fixed and incubated by the different primary antibodies (anti-FLAG for DRD1 and anti-LRRK2 (MJFF2) for LRRK2) and with Alexa647-conjugated secondary antibody (red) or Alexa488-conjugated secondary antibody (green) for DRD1 or LRRK2 respectively. Scale bars = 10μm. (C) Quantification of the average (mean ± SEM) of D1 puncta area from each image from two independent experiments showed in Fig 7B, 7D and 7E. **p<0,01; ***p<0,001. Two-way ANOVA and Bonferroni post test were used. (TIF) [file pone.0179082.s002.tif]

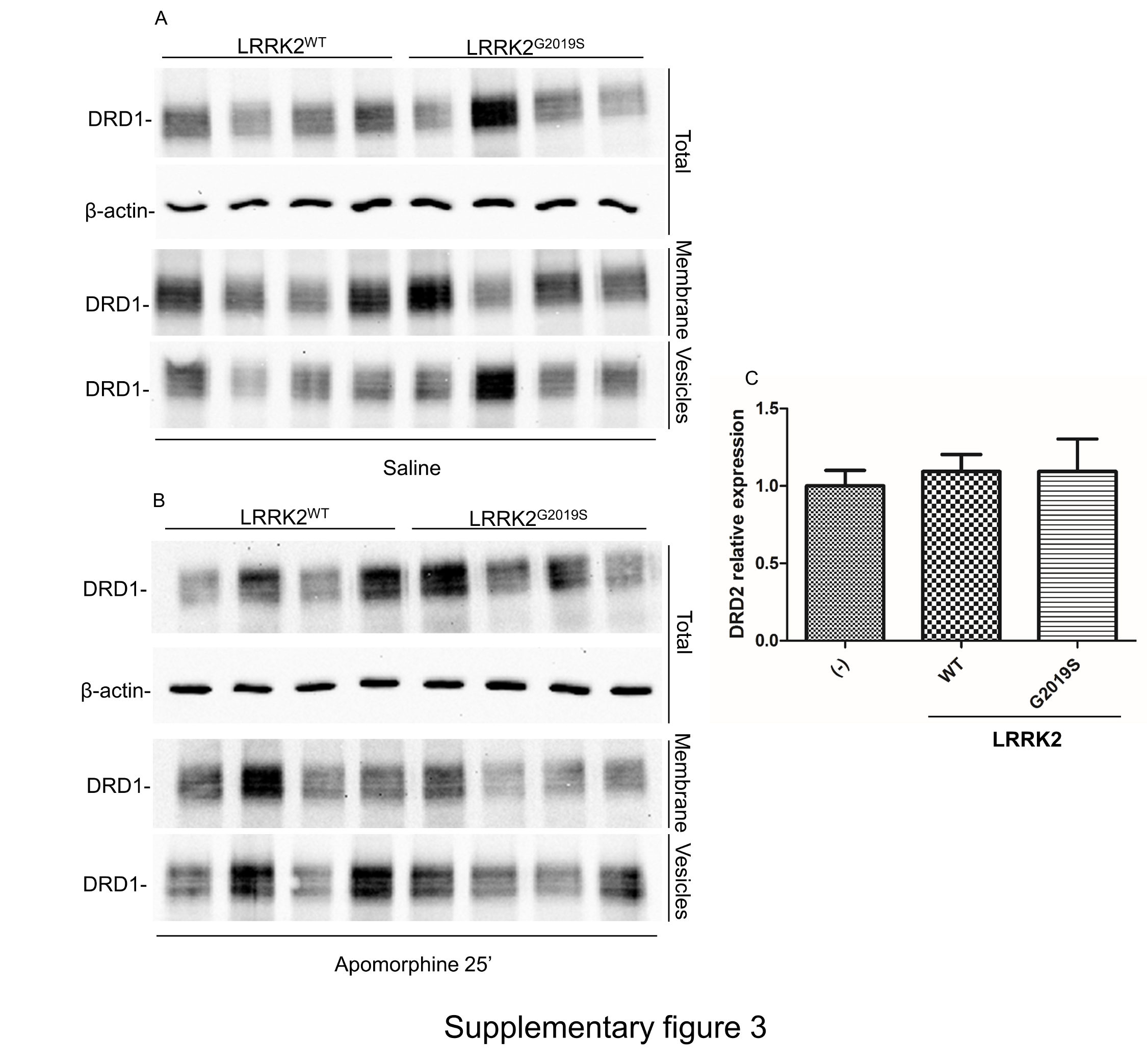

Supplement: S3 Fig — (A and B) Analysis by western blot of DRD1 protein level on total, membrane or vesicle protein fraction purified from striatum of WT or G2019S knock-in mice treated (B) or not (A) by apomorphine. (C) Analysis of DRD2 mRNA by real time PCR in the presence of WT or G2019S LRRK2 mutant in the same experimental conditions of Fig 4. (TIF) [file pone.0179082.s003.tif]
